# Supplementary material for: Scalable production of structurally colored composite films by shearing supramolecular composites of polymers and colloids
Source: Nat Commun. 2024 Feb 29;15:1874. doi: 10.1038/s41467-024-46237-4 (PMC10904808; doi:10.1038/s41467-024-46237-4)
Supplement: Supplementary file 1 — Supplementary Information [file 41467_2024_46237_MOESM1_ESM.pdf]

*Supplementary information for*

# Scalable production of structurally colored composite films by shearing supramolecular composites of polymers and colloids

Miaomiao Li[1], Bolun Peng[1], Quanqian Lyu[1], Xiaodong Chen[1], Zhen Hu[1], Xiujuan Zhang[1], Bijin Xiong[1], Lianbin Zhang[1,*], Jintao Zhu[1]

[1]State Key Laboratory of Material Processing and Die & Mould Technology and School of Chemistry and Chemical Engineering, Huazhong University of Science and Technology (HUST), Wuhan 430074, China.

E-mail: zhanglianbin@hust.edu.cn (L. Z.); Fax: (+86) 27 87543632

## This PDF file includes:

Supplementary Methods

Supplementary Discussions

Supplementary Tables

Supplementary References

## 1. Supplementary Methods

Synthesis of polystyrene (PS) colloids: PS colloids without carboxyl groups were also synthesized by emulsifier-free polymerization[1]. Briefly, 8.0 mL styrene and 400 mL water were mixed in a 500 mL flask. After de-oxygen, 0.28 g potassium persulfate was added to the above mixture, and the mixture was stirred for 6 h at 75 °C under $N_2$ atmosphere. Finally, the PS colloids were separated by centrifugation and washed with ethanol and water three times.

Synthesis of octadecyl-modified silica ($SiO_2$-C18) colloids: The synthesis of $SiO_2$-C18 colloids consists of two steps: synthesis and surface modification of $SiO_2$ colloids[2]. First, 100 mL ethanol, 7 mL $H_2O$, and 4 mL ammonium hydroxide were mixed. Then, 7 mL tetraethyl orthosilicate was added to the resulting mixture, and the mixture was stirred for 5 h at 30 °C. Then, 0.5 mL octadecyltrimethoxysilane was added, followed by a reaction at 40 °C for 12 h. Finally, the resultant products were thoroughly washed with ethanol three times by centrifugation.

## 2. Supplementary Discussions

### 2.1 Characterization of PS-COOH colloids synthesized by soap-free emulsion polymerization

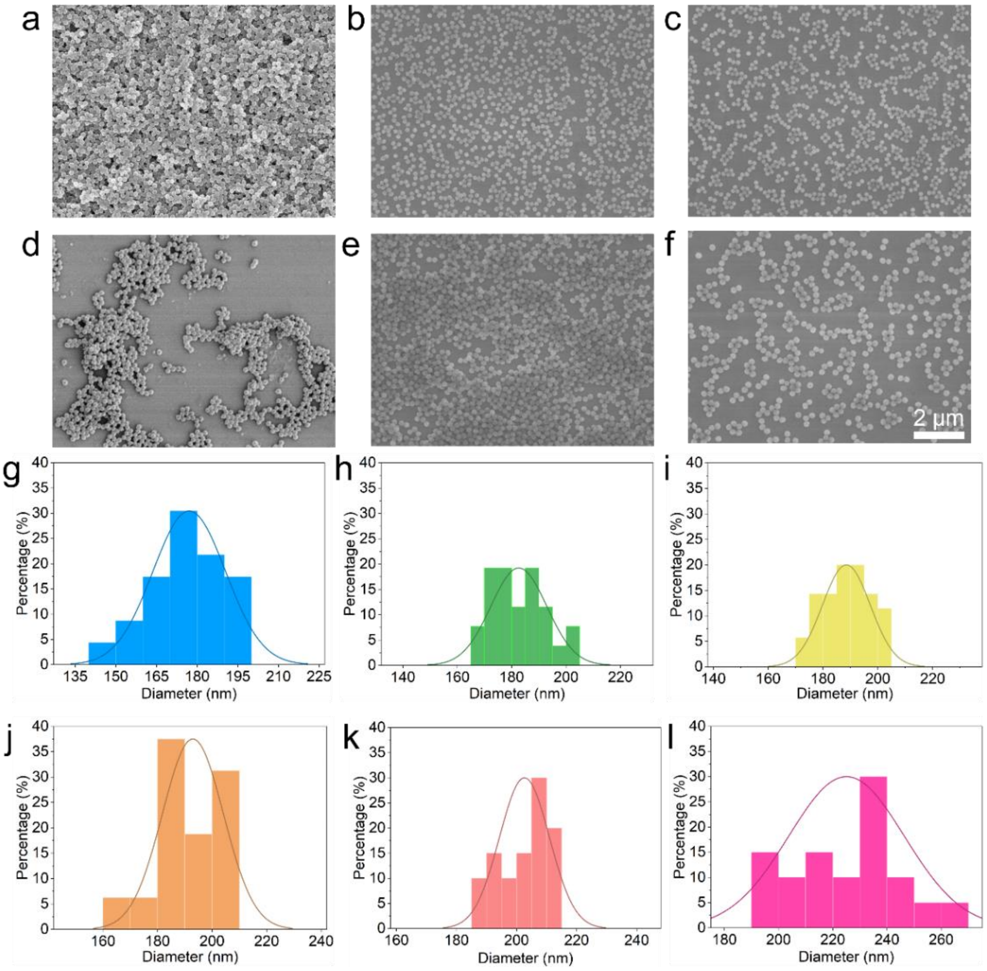


**Supplementary Fig. 1.** SEM images and the corresponding histograms of the size distribution of colloids with average diameters of 177 nm (**a, g**), 182 nm (**b, h**), 188 nm (**c, i**), 192 nm (**d, j**), 203 nm (**e, k**), and 225 nm (**f, i**).

## 2.2 Supramolecular interactions in the composites

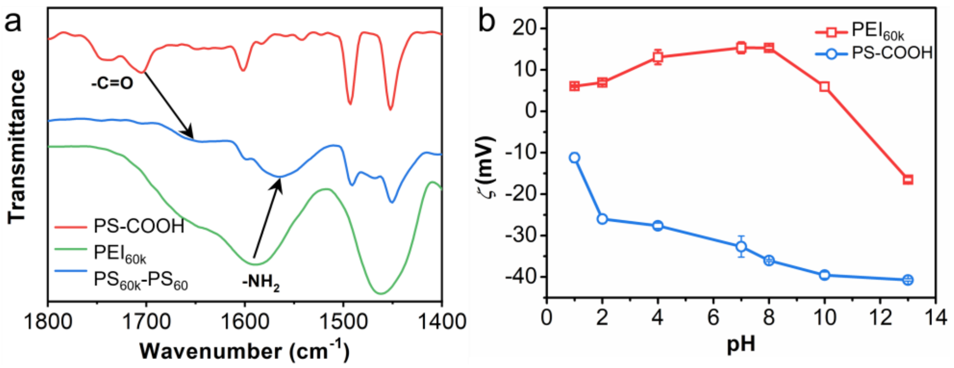


**Supplementary Fig. 2.** The supramolecular interactions between carboxylated polystyrene (PS-COOH) colloids and polyethyleneimine (PEI) in the PEI-PS composite. **a** FTIR spectra of PS-COOH colloid, PEI with $M_n$ of 60 kDa ($PEI_{60k}$), and the $PEI_{60k}$-$PS_{60}$ composite. **b** Zeta potential ( $\zeta$ ) of PS-COOH dispersion and $PEI_{60k}$ solution under different pH values. Error bars represent mean ± standard deviations. $n$ = 3 independent experiments.

**Supplementary Note 1:** To identify the interactions between PEI and PS-COOH, FTIR spectra of PS-COOH colloids, $PEI_{60k}$, and $PEI_{60k}$-$PS_{60}$ composite were investigated. In the FTIR spectra of PS-COOH and $PEI_{60k}$, the peaks at 1705 and 1590 $cm^{-1}$ correspond to the vibrational peaks of the carboxylic acid (-COOH) in PS-COOH and the amine groups ($-NH_2$) in $PEI_{60k}$, respectively. In the $PEI_{60k}$-$PS_{60}$ composite, these characteristic peaks were shifted to 1654 and 1564 $cm^{-1}$, respectively, indicating the hydrogen bonding between $PEI_{60k}$ and PS-COOH[3]. Moreover, the Zeta potential of $PEI_{60k}$ in near-neutral solutions was +15 mV, and the Zeta potential of PS-COOH colloids was -32 mV, indicating the electrostatic interaction between PEI and PS-COOH in the composite.

## 2.3 Effect of the dispersibility of colloids on shear-induced ordering

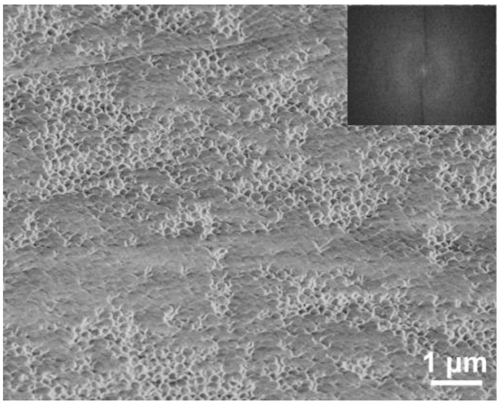


**Supplementary Fig. 3.** Scanning electron microscopy (SEM) image of the $PEI_{60k}$-$PS_{60}$ composite composed of $PEI_{60k}$ and PS-COOH colloids. Inset: the corresponding two-dimensional (2D) fast Fourier transform (FFT) image showing the homogeneous dispersion of PS-COOH colloids in the polymeric matrix.

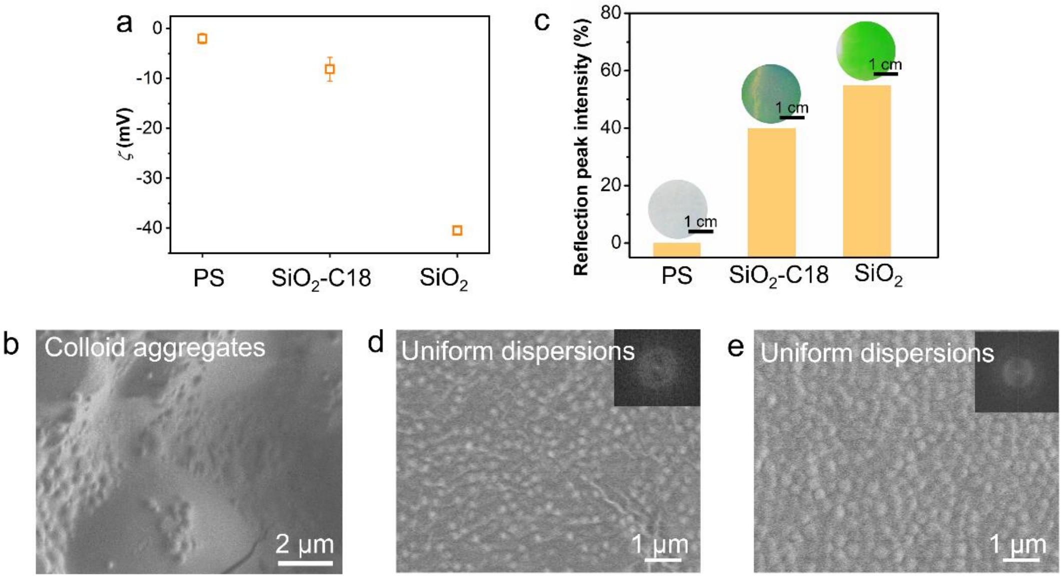


**Supplementary Fig. 4.** The dispersibility of colloids in the polymeric matrix and the corresponding shear-induced ordering effect. **a** Zeta potential ($\zeta$) of different colloid (PS, $SiO_2$-C18, and $SiO_2$) dispersions in near-neutral conditions. Error bars represent mean ± standard deviations. $n$ = 3 independent experiments. **b** SEM image of a composite constructed from PS colloids and $PEI_{60k}$. **c** Reflection peak intensity of composite films constructed from PS, $SiO_2$-C18, and $SiO_2$ colloids after shearing treatments. The reflection peak intensity is defined as the difference between the reflectivities at the peak and valley in the reflection spectrum. Insets: the corresponding photographs (sample diameter: 2.4 cm). **d**, **e** SEM images of composites constructed

from $SiO_2$-C18 and $SiO_2$ colloids with $PEI_{60k}$, respectively. Insets in (**d** and **e**): the corresponding 2D FFT images.

**Supplementary Note 2:** The Zeta potentials of as-prepared PS, $SiO_2$-C18, and $SiO_2$ colloids were -2, -8, and -42 mV, respectively (Supplementary Fig. 4a). Different composites were obtained by blending these colloidal dispersions with $PEI_{60k}$ solutions. The dispersibility of colloids in polymer matrices was observed using SEM imaging technique. PS colloids without surface charges exhibited significant aggregation in the polymeric matrix (Supplementary Fig. 4b). After shearing treatment, no noticeable structural color was observed in the resulting composite film (Supplementary Fig. 4c insets). In contrast, $SiO_2$-C18 and $SiO_2$ colloids were uniformly dispersed in the polymeric matrix (Supplementary Fig. 4d, e), which could be attributed to the supramolecular interactions between the colloids and the polymer that promote the uniform dispersion of the colloids in the polymeric matrix. Upon shearing treatments, these composite films composed of $SiO_2$-C18 and $SiO_2$ colloids exhibited remarkable structural colors with reflection peak intensities of 40% and 55% (Supplementary Fig. 4c), respectively. These results revealed that the uniform dispersion of colloids in the polymeric matrix was a prerequisite for shear-induced ordering. Furthermore, we observed that appropriate supramolecular interactions between the polymer and the colloid significantly improved the shear-induced ordering effects. This can be explained by the fact that the appropriate supramolecular interactions might facilitate momentum transfer between the colloid and the polymer[4], resulting in a well-ordered colloidal arrangement.

## 2.4 The shear thinning performance of the supramolecular composite

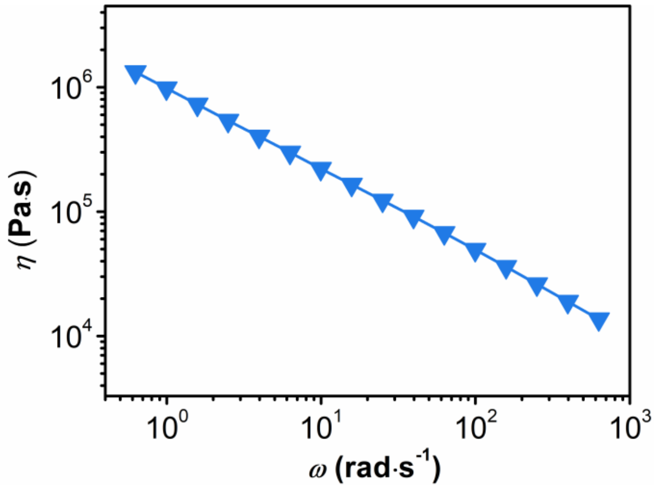


**Supplementary Fig. 5.** The shear thinning properties of supramolecular composite. The complex viscosity ($\eta$) of PEI$_{60k}$-PS$_{60}$ composite as a function of the angular frequency ($\omega$). The complex viscosity was determined in a rotational rheometer. The complex viscosity of the supramolecular composite decreased by ~ 2-3 orders of magnitude during the oscillation angular frequencies from 0.628 to 628 rad s$^{-1}$, implying a strong shear thinning performance of the supramolecular composite.

## 2.5 Optimization of the processing parameters

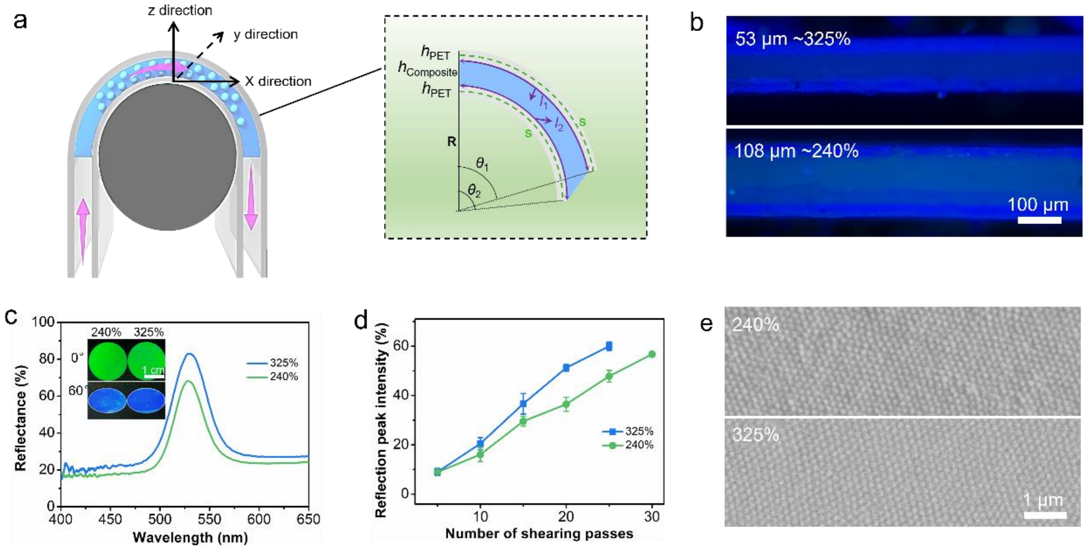


**Supplementary Fig. 6.** Effect of the shear strain ($\gamma$) on the shear-induced ordering. **a** Illustration showing shear strain generated by bending the sandwiched film onto a fixed rod and the corresponding *x*, *y*, and *z* directions. The relative shifts between the top and bottom polyethylene terephthalate (PET) films of length *s* indicate the strain difference during shearing treatment. **b** Optical microscopy images showing the thickness of the

composite film inside two PET sheets. **c** Reflection spectra of $PEI_{60k}$-$PS_{60}$ films under shear strains of 240% and 325% after 25 shearing passes (rod diameter: 3 mm, load: 5 N). Insets in (c): the corresponding photographs at viewing angles of 0° and 60° (sample diameter: 1.9 cm). **d** Reflection peak intensity of $PEI_{60k}$-$PS_{60}$ films at different shear strains as a function of the number of shearing passes. Error bars represent mean ± standard deviations. *n* = 3 independent experiments. **e** SEM images of $PEI_{60k}$-$PS_{60}$ films under shear strains of 240% (top panel) and 325% (down panel) after 25 shearing passes.

**Supplementary Note 3:** To compare the ordering effects produced by shearing different supramolecular composites, the relevant processing parameters, including the thickness of the supramolecular composite ($h_{\mathrm{Composite}}$), the diameter of the rod ($D$), and the load ($G$), were optimized individually while other parameters were held constant. In the bending-induced shearing treatment process, bending the sandwiched film around the rod generates a strong shearing force inside the composite film parallel to the surface[5]. The corresponding schematic representation is shown in Supplementary Fig. 6a. $R$ is the radius of the rod, $s$ is the unstretched length of the top and bottom PET films (green dotted lines), and $l_1$ and $l_2$ are the lengths of the top and bottom surfaces of the composite film in contact with two PET films of length $s$ (purple solid lines), respectively. $h_{\mathrm{Composite}}$ and $h_{\mathrm{PET}}$ are the thicknesses of the composite film and PET, respectively. $\theta_1$ and $\theta_2$ are the angles at the center of the rod between the rod midpoint and the last contact points of the top and the bottom PET films of length $s$, respectively. $\Delta l$ is the displacement difference of the composite film between the top and the bottom PET planes during the shearing process. In this case, $\Delta l = l_2 - l_1 = (\theta_2 - \theta_1)(R + h_{\mathrm{PET}})$ and $s = (R + \frac{1}{2}h_{\mathrm{PET}})\theta_2 = (R + h_{\mathrm{Composite}} + \frac{3}{2}h_{\mathrm{PET}})\theta_1$. Therefore, the shear strain $\gamma$ can be calculated by Supplementary Equation 1[5]:

$$\gamma = \frac{\Delta l}{h_{\mathrm{Composite}}} = \frac{R + h_{\mathrm{PET}}}{h_{\mathrm{Composite}}}(\theta_2 - \theta_1) \approx \left(1 + \frac{h_{\mathrm{PET}}}{h_{\mathrm{Composite}}}\right)\theta_2 \qquad \mathbf{(1)}$$

To investigate the effect of shear strain ($\gamma$) on the shear-induced colloidal ordering, we prepared sandwiched films with thicknesses of 222 and 167 μm (Supplementary Fig. 6b), using a hot-press apparatus by adjusting the temperature and pressure during the process. The corresponding thicknesses of the resulting composite films were 108 and 53 μm. When the sandwich film was bent 180° around the rod, i.e., $\theta_2 = \frac{\pi}{2}$, the shear strains generated in the composite films with thicknesses of 108 and 53 μm can be

calculated to be 240% and 325%, respectively, according to Supplementary Equation 1. For the same number of shearing passes (25 passes), we noted that the green appearance of the composite films did not show significant differences in brightness when the shear strain increased from 240% to 325%. The corresponding peak intensities increased by less than 10% (Supplementary Fig. 6c). Furthermore, the relationship between the peak intensity and the number of shearing passes showed no significant difference under shear strains of 240% and 325% (Supplementary Fig. 6d), which was further supported by observations from SEM images (Supplementary Fig. 6e). These results indicate that for current supramolecular composites, a shear strain of 240% induced by bending is sufficient to achieve shear-induced ordering. Notably, for larger shear strains, there were fewer stacked layers in the composite film, which would be detrimental to the overall reflectivity of the film. In this case, we employed a composite film with a thickness of ~ 100 μm for shearing treatment.

**Supplementary Note 4:** The shear strain rate ($\dot{\gamma}$) during the shearing process can be calculated by Supplementary Equation 2.

$$\dot{\gamma} = \frac{v}{h_{\mathrm{Composite}}} \quad (2)$$

Where $v$ is the winding speed rate of the composite film during shearing treatment (1.8 m·min$^{-1}$), therefore, for a composite film with a thickness of 108 μm, $\dot{\gamma}$ can be calculated as 278 s$^{-1}$.

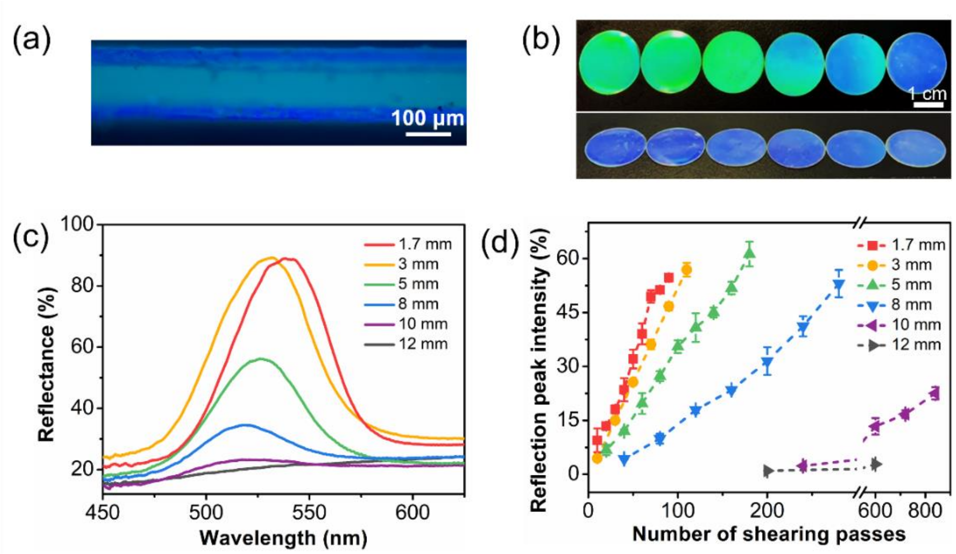


**Supplementary Fig. 7.** Optimization of the diameter of the rod. **a** Optical microscopy image of the cross section of the sandwiched film, including two PET films and a

composite film. **b**, **c** Photographs and reflection spectra of $PEI_{60k}$-$PS_{60}$ films produced under 100 shearing passes with a fixed load (*G*: 2 N) and the thickness of the $PEI_{60k}$-$PS_{60}$ film ($h_{Composite}$: ~ 94.4 μm) but varying rods with diameters of 1.7, 3, 5, 8, 10, and 12 mm. **d** Reflection peak intensity of $PEI_{60k}$-$PS_{60}$ films produced by using rods with different diameters as a function of the number of shearing passes. Error bars represent mean ± standard deviations. $n = 3$ independent experiments.

**Supplementary Note 5:** The rod diameter (*D*) significantly affects the compressive stress of the film, thereby affecting the shear-induced ordering effects of colloids[6]. To obtain high-quality structural colors, we further optimized the rod diameter. Specifically, the sandwiched film was bent along rods with varying diameters under a fixed load of 2 N, while the reflective spectrometer recorded in situ the change in intensity of the reflection peaks at different shearing passes. Taking a $PEI_{60k}$-$PS_{60}$ film with a thickness of ~ 94.4 μm as an example (Supplementary Fig. 7a), when the rod diameter increased from 1.7 to 12 mm, the brightness of the green appearance of the $PEI_{60k}$-$PS_{60}$ film decreased significantly and gradually lost its angular dependence at the same number of shearing passes (Supplementary Fig. 7b). Correspondingly, the reflection peak intensity decreased from ~ 65% to ~ 0% (Supplementary Fig. 7c). These results imply that reducing the rod diameter promoted shear-induced colloidal ordering. Notably, when the rod diameter was reduced from 3 to 1.7 mm, there was no significant change in the peak intensity of the corresponding composite film, indicating no significant improvement in its optical quality. This may be due to the fact that a smaller diameter rod generated a larger average compressive stress within the composite film at a given load, leading to more defects. The average compressive stress generated in the composite film on rods with different diameters during shearing treatment can be calculated using Supplementary Equation 3. These results suggested that rods with diameters of ~ 3-8 mm are suited for the shearing processing of current supramolecular composites (Supplementary Fig. 7d).

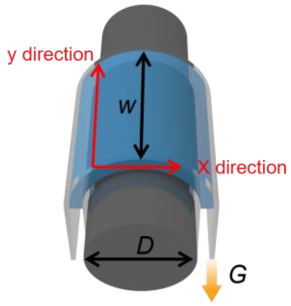


**Supplementary Fig. 8.** Illustration showing compressive stress generated by bending the sandwich film onto a rod under a specific load *G*.

As depicted in Supplementary Fig. 8, the average compressive stress $\bar{P}$ can be calculated by Supplementary Equation 3.

$$\bar{P} = \frac{G}{S'} = \frac{G}{w \times D} \quad \textbf{(3)}$$

Where $G$ is the weight of the load, and $S'$ is the projected area of the contact surface between the rod and the sandwich film along the *z*-direction (i.e., thickness direction, depicted in Supplementary Fig. 6a). *w* is the width of the sandwiched film along the *y*-direction (30 mm). Therefore, for a composite film sheared under different rods with diameters of 1.7, 3, 5, 8, 10, and 12 mm, $\bar{P}$ can be calculated as 39, 22, 13, 8.3, 6.7, and 5.5 kPa.

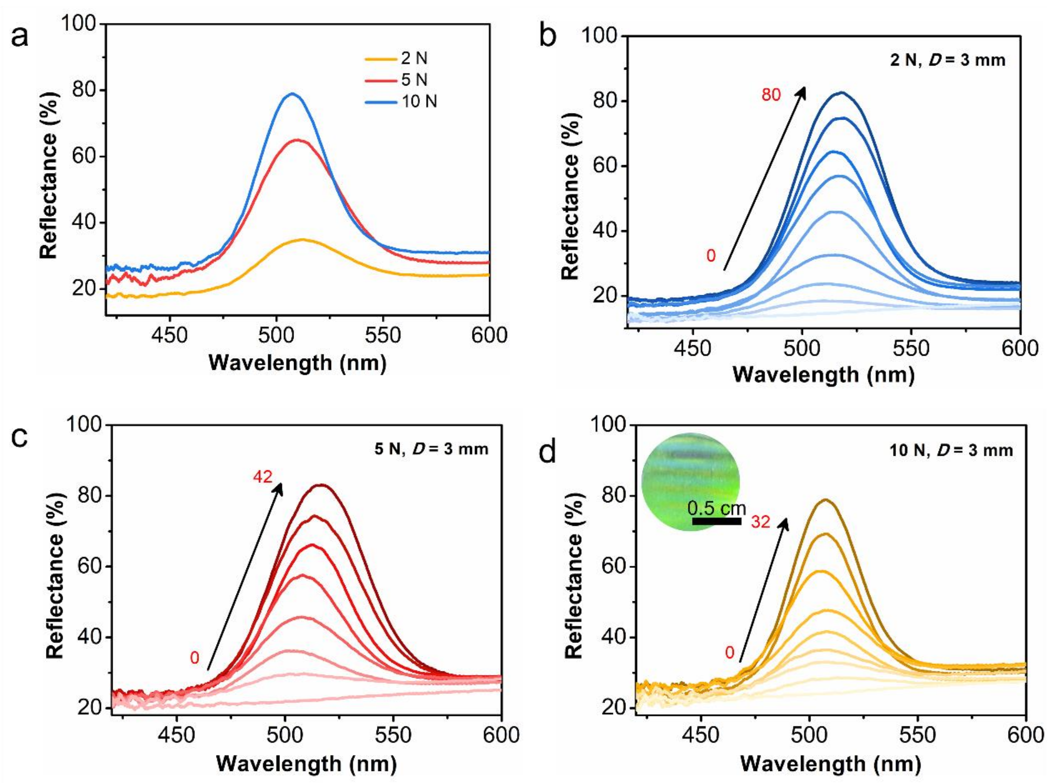


**Supplementary Fig. 9.** Optimization of the load ($G$) on the shear-induced ordering. **a** Reflection spectra of $PEI_{60k}$-$PS_{60}$ films produced under 30 shearing passes with a fixed rod diameter ($D$, 3 mm) and composite film thickness ($h_{Composite}$: ~ 100 μm) but with varying loads of 2 N, 5 N, and 10 N. **b**, **c**, **d** The reflection spectra of $PEI_{60k}$-$PS_{60}$ films sheared under loads of 2 N, 5 N, and 10 N as a function of the number of shearing passes. Inset in (d): the photograph of the $PEI_{60k}$-$PS_{60}$ film produced under a load of 10 N.

**Supplementary Note 6:** Different from rod diameter, the load affects the compressive stress of the film while also affecting the shear stress generated inside the composite film during shearing treatment. When the shear stress generated by the load is higher than the yield stress of the composite, the arrangement of colloids could be altered during shearing treatments[7]. Therefore, to obtain high-quality structural colors, the load should be further optimized. In bending-induced shearing treatment, the shear stress generated by the load can be calculated by Supplementary Equation 4.

$$\sigma = \frac{G}{w \times \Delta l} \approx \frac{G}{w \times (h_{\mathrm{Composite}} + h_{\mathrm{PET}}) \times \theta_2} \quad \mathbf{(4)}$$

To investigate the effect of the load on shear-induced colloidal ordering, b-$PEI_{60k}$-$PS_{60}$ films were sheared under loads of 2, 5, and 10 N, and the corresponding shear

stresses were calculated to be 0.29, 0.71, and 1.43 MPa, respectively, according to Supplementary Equation 4. Other processing parameters were fixed, including a rod diameter of ~ 3 mm, a composite film thickness of ~ 100 μm, and a PET thickness of ~ 57 μm. As shown in Supplementary Fig. 9a, compared to the load of 2 and 5 N, the $PEI_{60k}$-$PS_{60}$ film showed a higher reflection peak intensity under the high load of 10 N during shearing. This can be attributed to high shear stress induced by the high load, which promoted polymer chain stretching and colloidal momentum transfer[4,8]. In addition, by quantitatively studying the variation in the reflection peak intensity versus the number of shearing passes under different loads, we found that 80 shearing passes were required to construct structurally colored composite films (SCCFs) with a reflection peak intensity of up to 55% at a load of 2 N. In comparison, only 42 shearing passes were required at a load of 5 N, and 32 shearing passes were needed for a load of 10 N (Supplementary Fig. 9b, c, and d). These results indicate that the magnitude of the load is critical for obtaining SCCFs with high-quality structural colors. It is worth noting that when the load reached 10 N, defects appeared in the composite films during shearing treatment (Supplementary Fig. 9d), which may be related to the mechanical strength of the composite. Therefore, a load of 5 N is optimal for constructing composite films with high-quality structural colors.

**2.6 Viscoelastic properties of neat PEIs with different molecular weights and corresponding composites**

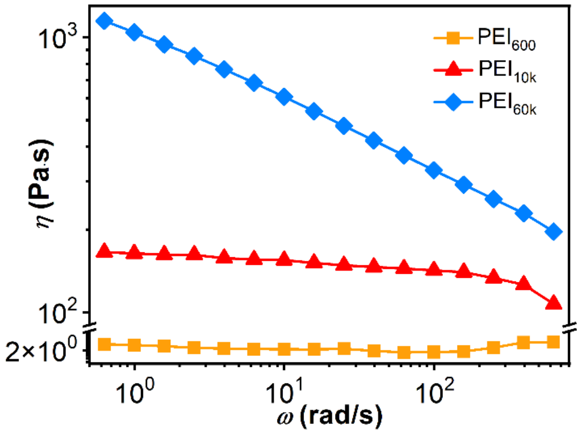


**Supplementary Fig. 10.** The viscosity of $PEI_{600}$, $PEI_{10k}$, and $PEI_{60k}$ as a function of angular frequency ($\omega$). The measurement was performed with a strain amplitude of 0.1%.

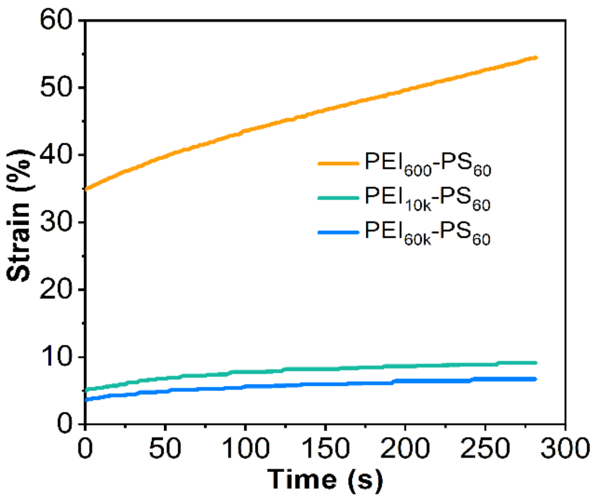


**Supplementary Fig. 11.** Time dependence of the strain, $\varepsilon$, for $PEI_{600}$-$PS_{60}$, $PEI_{10k}$-$PS_{60}$, and $PEI_{60k}$-$PS_{60}$ composites. The external stress value was 20 kPa.

## 2.7 Shear-induced ordering effects of composites composed of PEG-*b*-PPG-*b*-PEG with different $M_n$

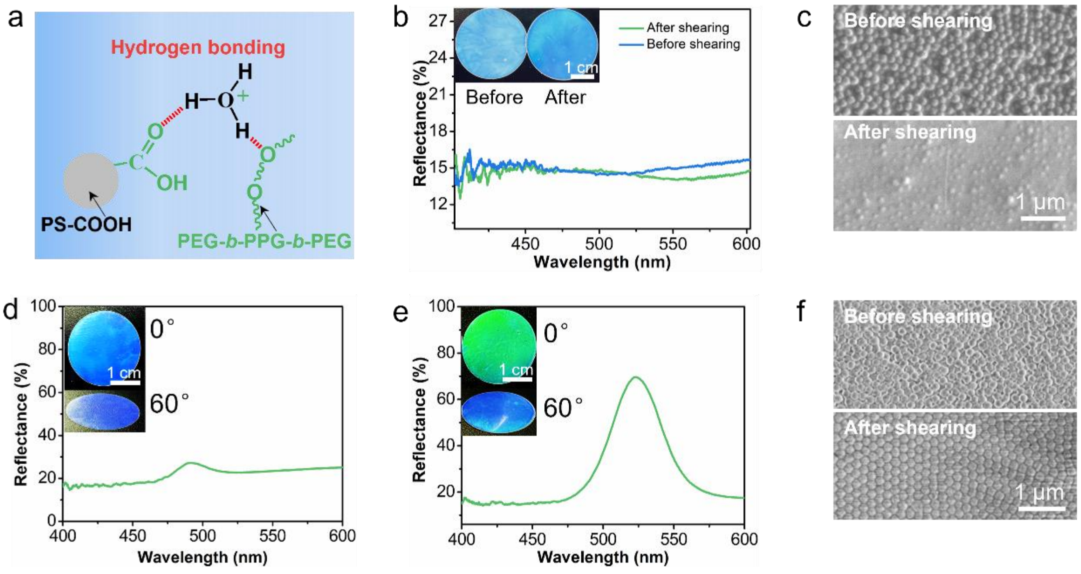


**Supplementary Fig. 12. a** Scheme showing the hydrogen bonding interaction between poly(ethylene glycol)-*block*-poly(propylene glycol)-*block*-poly(ethylene glycol) triblock copolymers (PEG-*b*-PPG-*b*-PEG) and PS-COOH. Different PEG-*b*-PPG-*b*-PEG with $M_n$ of 2.9 kDa, 8.6 kDa, and 14.6 kDa were purchased from Beijing Innochem Corporation Ltd. **b**, **c** Reflection spectra and SEM images of the composite film composed of PS-COOH ($\varphi_{PS}$: 60%) and PEG-*b*-PPG-*b*-PEG with an $M_n$ of 2.9 kDa before and after shearing treatments. Insets in (b): the corresponding photographs of the composite film composed of PEG-*b*-PPG-*b*-PEG with $M_n$ of 2.9 kDa before (left) and after (right) shearing treatments (sample diameter: 3.0 cm). **d**, **e** Reflection spectra of

composite films consisting of PEG-*b*-PPG-*b*-PEG with $M_n$ of 8.6 kDa and 14.6 kDa after shearing treatments. Insets in (d and e): the corresponding photographs at viewing angles of 0° (top) and 60° (bottom) (sample diameter: 2.4 cm). **f** SEM images of the composite film composed of PEG-*b*-PPG-*b*-PEG with $M_n$ of 14.6 kDa before and after shearing treatments.

**Supplementary Note 7:** To investigate the universality of the shear-induced ordering mechanism associated with $M_n$, PEG-*b*-PPG-*b*-PEG with different $M_n$ and PS-COOH was chosen because the ether groups on the polymer chains can form hydrogen bonds with the carboxyl groups on the surface of PS-COOH[10] (Supplementary Fig. 12a). It is known that linear polyether chains become entangled at a critical $M_n$ of ~ 6-7 kDa[11]. Therefore, neat PEG-*b*-PPG-*b*-PEG with $M_n$ of 2.9 kDa, 8.4 kDa, and 14.6 kDa exhibited viscous liquid behavior with nonentanglement, slight entanglement, and significant entanglement, respectively. These PEG-*b*-PPG-*b*-PEG polymers were chosen to construct supramolecular composites, and we used photography, reflection spectroscopy, and SEM imaging to investigate shear-induced ordering effects. After shearing treatment, the composite consisting of PEG-*b*-PPG-*b*-PEG with $M_n$ of 2.9 kDa showed no obvious change in appearance and colloidal structure compared to that before shearing, with angle-independent structural colors and low-ordered colloidal structures (Supplementary Fig. 12b, c). In contrast, the composite consisting of PEG-*b*-PPG-*b*-PEG with $M_n$ of 8.4 kDa showed a weak and low angle-dependent structural color (Supplementary Fig. 12d), while the composite consisting of PEG-*b*-PPG-*b*-PEG with $M_n$ of 14.6 kDa showed a strong angle-dependent structural color and a highly ordered colloidal structure (Supplementary Fig. 12e, f). These results suggested that chain entanglement of polymers in the supramolecular composite can promote the colloid to obtain sufficient momentum to form ordered colloidal arrangements. This result is consistent with PEIs and implies the universality of the shear-induced ordering mechanism associated with $M_n$.

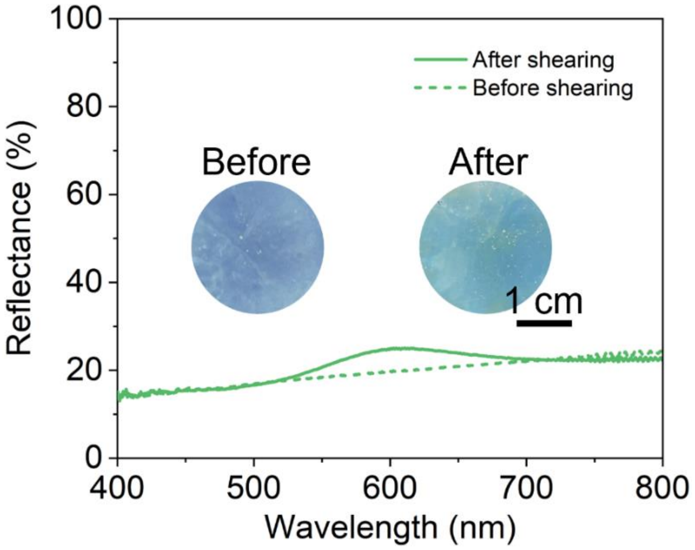


**Supplementary Fig. 13**. Reflection spectra of the SCCFs with PEI molecular weight of 2000 kDa before and after shearing treatments. Inserts: the corresponding photographs.

**2.8 Effect of colloid volume fraction on the optical and mechanical properties of supramolecular composites**

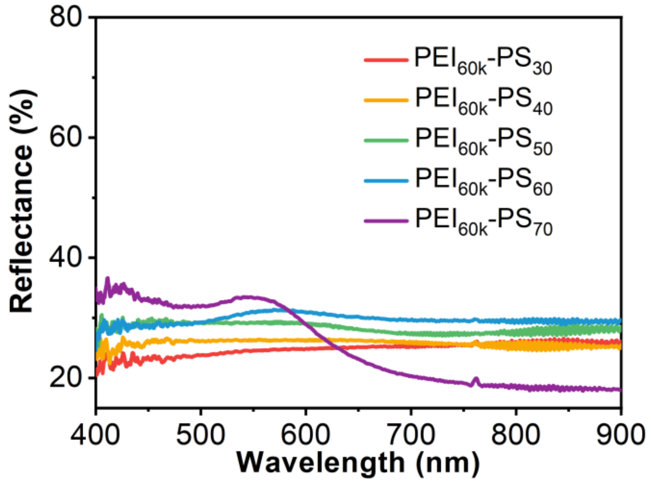


**Supplementary Fig. 14.** Reflection spectra of composite films composed of PEI with an $M_n$ of 60 kDa but varying volume fractions of PS-COOH.

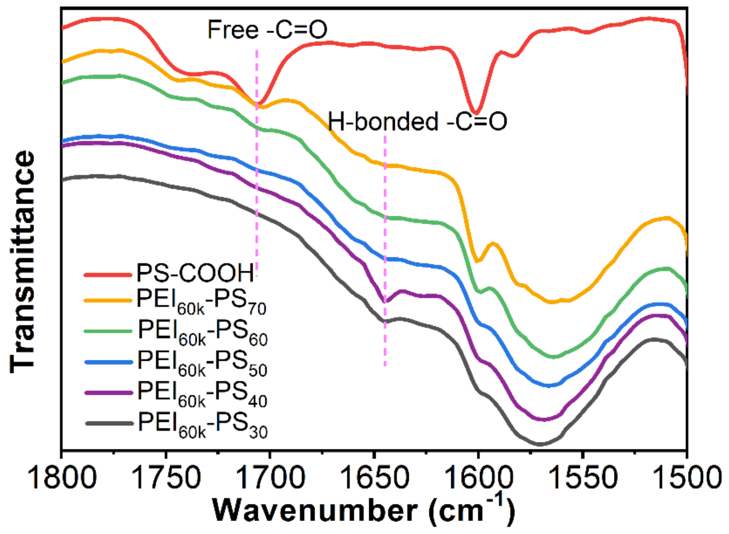


**Supplementary Fig. 15.** FTIR spectra of PS-COOH and the composites composed of PEI with an $M_n$ of 60 kDa but varying volume fractions of PS-COOH.

**Supplementary Note 8:** To gain insights into the constraints of polymer chains in supramolecular composites composed of different colloidal volume fractions, we prepared a series of supramolecular composites with $\varphi_{PS}$ of 30%, 40%, 50%, 60%, and 70%. We investigated the interactions between colloids and the polymers using FTIR spectroscopy. Compared with the PS-COOH colloids, the -C=O adsorption band in the composites exhibited splitting into two peaks (Supplementary Fig. 15). These peaks corresponded to the free and H-bonded -C=O on the PS-COOH in the composites[1]. We observed that as the volume fraction of PS-COOH increased, the peak of the free -C=O gradually appeared, while the H-bonded -C=O gradually redshifted. When the volume fraction of PS-COOH was or exceeded 60%, i.e., for $PEI_{60k}$-$PS_{60}$ and $PEI_{60k}$-$PS_{70}$ composites, free -C=O appeared in the composites, suggesting that sufficient carboxyl groups were exposed on the PS-COOH surface. Therefore, we can infer that, in this case, all PEIs were bound to the PS-COOH surface through supramolecular adsorption. When the volume fraction of PS-COOH was below 60%, i.e., for $PEI_{60k}$-$PS_{30}$, $PEI_{60k}$-$PS_{40}$, and $PEI_{60k}$-$PS_{50}$ composites, all free -C=O disappeared, indicating the presence of unabsorbed free PEI interspersing in the polymeric matrix[12]. These observations provide valuable insights for subsequent analysis of the influence of colloidal volume fraction on shear-induced ordering effects.

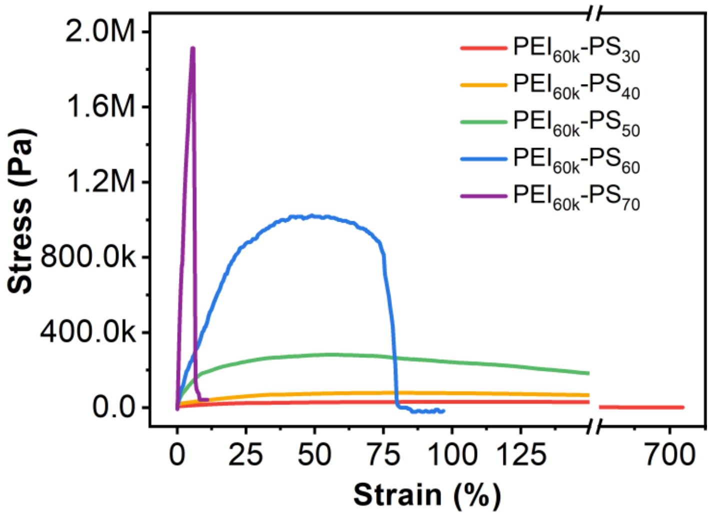


**Supplementary Fig. 16.** Stress–strain curves of $PEI_{60k}$-$PS_{30}$, $PEI_{60k}$-$PS_{40}$, $PEI_{60k}$-$PS_{50}$, $PEI_{60k}$-$PS_{60}$, and $PEI_{60k}$-$PS_{70}$ composites under a stretching rate of 50 mm·min$^{-1}$.

## 2.9 Colloidal arrangement and optical properties of SCCFs

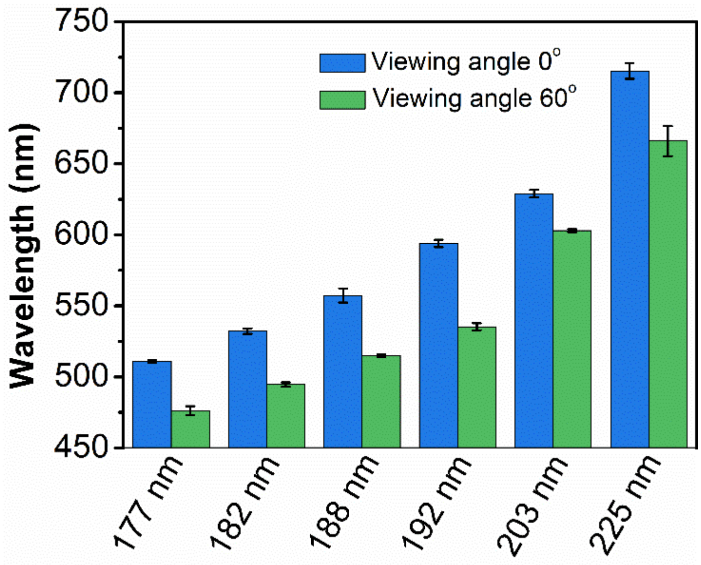


**Supplementary Fig. 17.** Reflection wavelengths of SCCFs with different colors constructed from colloids with different diameters at viewing angles of 0° and 60°. The colloid sizes used for preparing different SCCFs from left to right are 177, 182, 188, 192, 203, and 225 nm. Error bars represent mean ± standard deviations. $n$ = 3 independent experiments.

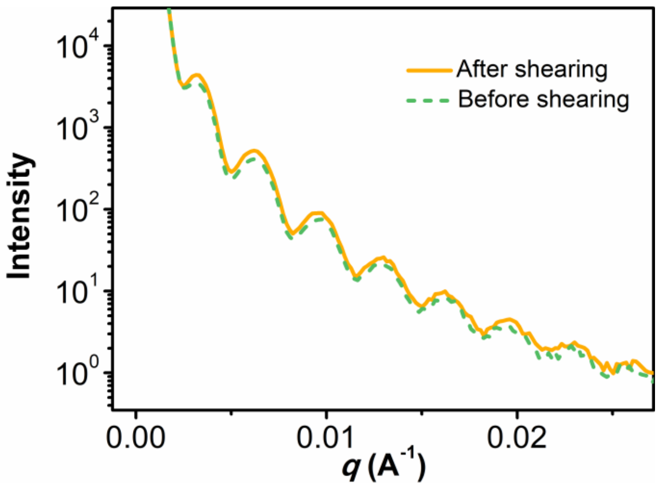


**Supplementary Fig. 18.** The one-dimensional (1D) small-angle X-ray scattering (SAXS) curves of the $PEI_{60k}$-$PS_{60}$ film before and after shearing treatments. SAXS spectroscopy was measured using a Xeuss 2.0 system (Xenocs France).

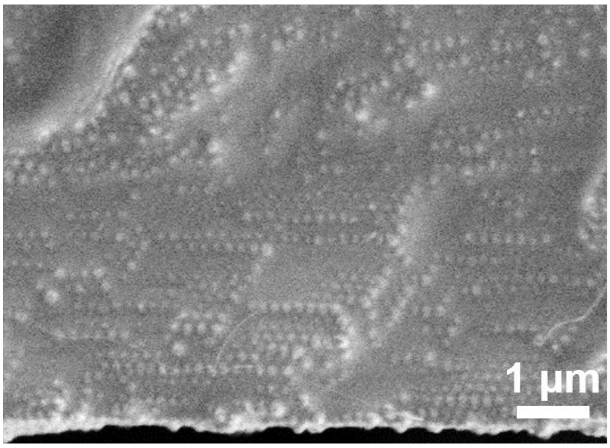


**Supplementary Fig. 19.** Cross-sectional SEM image of $PEI_{60k}$-$PS_{60}$ film with insufficient shearing treatment.

## 2.10 Effect of water on the processing performance

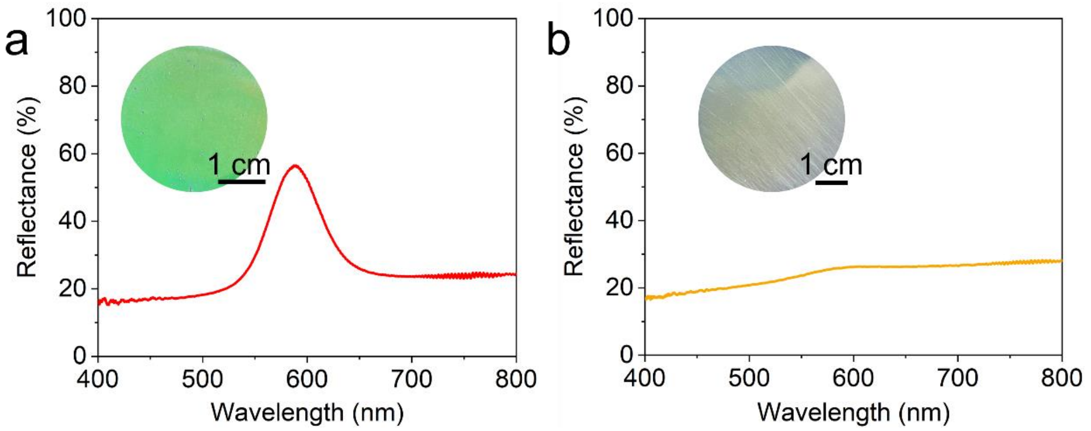


**Supplementary Fig. 20.** The reflection spectra of SCCF films with water contents of 25 wt.% (**a**) and 30 wt.% (**b**) after sufficient shearing treatment. Insets: corresponding photographs.

**Supplementary Note 9:** To ensure that the ordered colloidal arrangments generated by shearing treatment in these composites could be maintained, the thermal motion of PS-COOH in $PEI_{60k}$-$PS_{60}$ composites with different water contents was evaluated by the diffusion coefficient ( $D_0$ ) according to Supplementary Equation 5[2].

$$D_0 = \frac{kT}{6\pi\eta r} \quad \ldots\ldots (5)$$

Where $k$ is Boltzmann's constant, $T$ is the temperature, $\eta$ is the viscosity of the medium surrounding the colloids, and $r$ is the radius of PS-COOH. Due to the hydrogen bonding and electrostatic interactions between PS-COOH and PEI, the complex viscosity of the composite is used here to evaluate the thermal motion of PS-COOH. The complex viscosities of the composites with water contents of 10 wt.%, 15 wt.%, and 20 wt.% were determined by viscosity measurements to be $5.37\times10^5$, $3.10\times10^5$, and $0.817\times10^5$ Pa·s, respectively. Therefore, $D_0$ of the colloids in the composites with water contents of 10 wt.%, 15 wt.%, and 20 wt.% can be calculated as $4.06\times10^{-21}$, $7.03\times10^{-21}$, and $2.67\times10^{-20}$ $m^2\cdot s^{-1}$, respectively, indicating that these ordered colloidal arrangements produced by shearing were well fixed in these high-viscosity composites even after the removal of external forces.

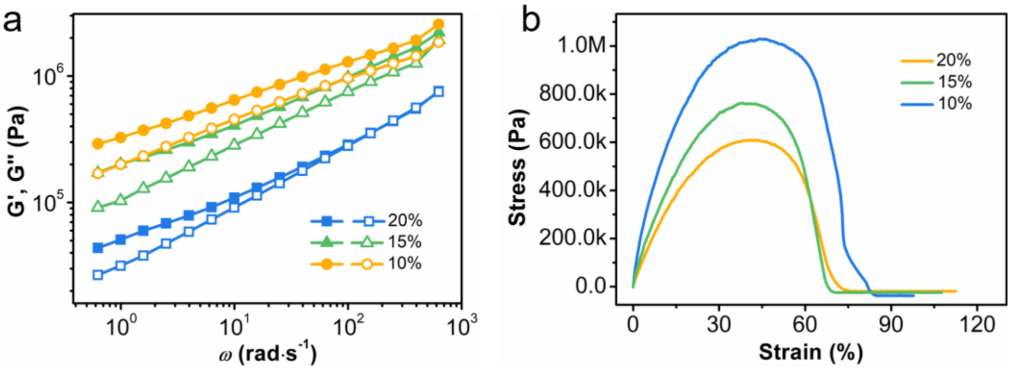


**Supplementary Fig. 21.** Effect of water content on the mechanical properties of supramolecular composites. **a** Dynamic shear moduli (G', solid; G'', open) of $PEI_{60k}$-$PS_{60}$ composites with water contents of 10 wt.%, 15 wt.%, and 20 wt.% as a function of frequency. **B** Stress–strain curves of $PEI_{60k}$-$PS_{60}$ composites with different water contents under a stretching rate of 50 mm·min$^{-1}$.

**Supplementary Note 10:** The mechanical properties of $PEI_{60k}$-$PS_{60}$ composites with different water contents under shearing were examined by frequency-dependent rheology and uniaxial tensile. As the water content increased from 10 wt.% to 15 wt.% and 20 wt.%, the storage modulus of these composites at an angular frequency of 0.628 rad·s$^{-1}$ decreased from 291 to 172 and 43 kPa (Supplementary Fig. 21a). This can be attributed to the fact that water promotes the dissociation of hydrogen bonding and electrostatic interactions, reducing the binding of polymer chains by supramolecular adsorption[13]. This result was confirmed by the decrease in Young's modulus and the increase in the tensile strain of the composites with increasing water content in the stress–strain curves (Supplementary Fig. 21b).

## 2.11 Effect of temperature on the processing performance

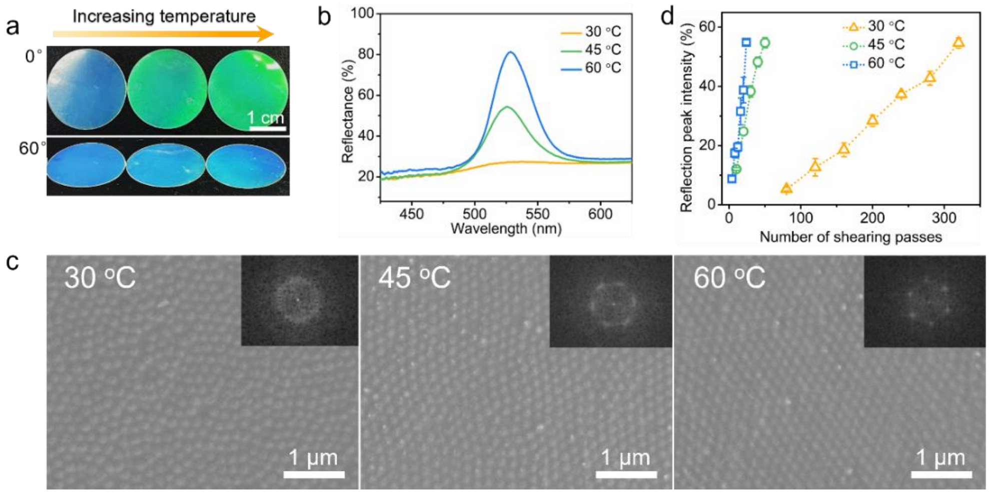


**Supplementary Fig. 22. A, b, c** Photographs, reflection spectra, and SEM images of the $PEI_{60k}$-$PS_{60}$ films after 20 shearing passes at 30, 45, and 60 °C (sample diameter: 2.2 cm). Insets in (c): the corresponding 2D FFT images. **d** Reflection peak intensity of the $PEI_{60k}$-$PS_{60}$ films with different processing temperatures as a function of the number of shearing passes. Error bars represent mean ± standard deviations. $n = 3$ independent experiments.

**Supplementary Note 11:** To investigate the effect of temperature on the processing performance of the supramolecular composite, the $PEI_{60k}$-$PS_{60}$ composites were sheared at processing temperatures of 30, 45, and 60 °C. By observing the photographs and changes in the reflection peak intensity, we found that by increasing processing temperatures from 30 to 45 and 60 °C, the resulting b-$PEI_{60k}$-$PS_{60}$ composite films exhibited a more intense green appearance with increased brightness under the same number of shearing passes (Supplementary Fig. 22a, b). This means that the optical quality of the composite film improved significantly with increasing processing temperature. Correspondingly, the ordering of colloidal arrangement increased with processing temperature (Supplementary Fig. 22c). To construct SCCF with a reflection peak intensity up to 55%, 320 shearing passes at 30 °C were required. In contrast, only 50 shearing passes at 45 °C and 24 shearing passes at 60 °C were needed (Supplementary Fig. 22d). The productivity at 60 °C was 13 times higher than that at 30 °C for the same shear condition. The significant reduction in the number of shearing passes and the increased productivity by the slight increase in processing temperature demonstrates the unique processing advantages of the supramolecular composite for the construction of high-quality SCCFs.

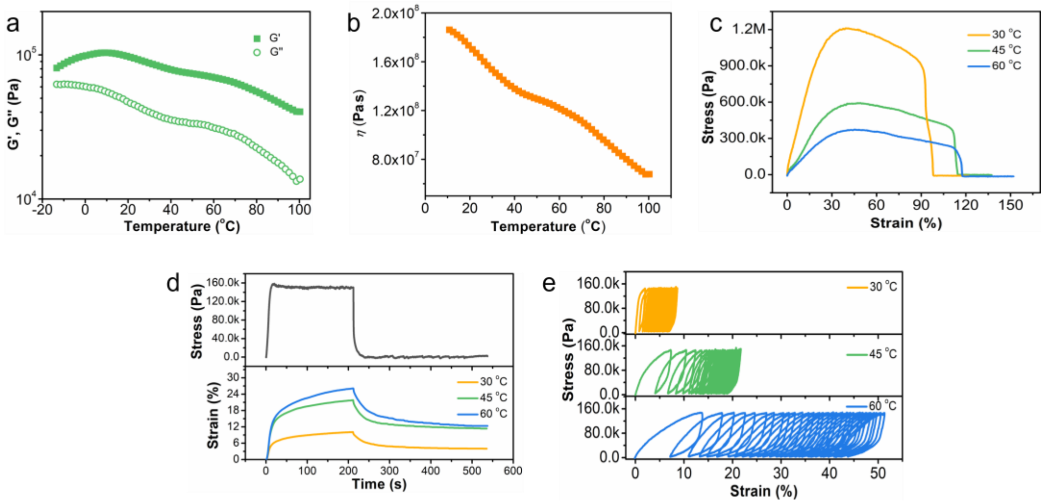


**Supplementary Fig. 23.** Effect of processing temperature on the mechanical properties of supramolecular composites. **a**, **b** Dynamic shear moduli (G', solid; G'', open) and viscosity of $PEI_{60k}$-$PS_{60}$ composites as a function of temperature with a heating rate of 5 °C·min$^{-1}$. The measurements were performed with an oscillation frequency of 0.1 Hz and a strain amplitude of 0.1%. **c** Stress–strain curves of $PEI_{60k}$-$PS_{60}$ composites at processing temperatures of 30, 45, and 60 °C under a stretching rate of 50 mm·min$^{-1}$. **d** Time dependence of the strain, $\varepsilon$, for the $PEI_{60k}$-$PS_{60}$ composite under different processing temperatures. The external stress value was 160 kPa. **e** Cyclic stress–strain curves of the $PEI_{60k}$-$PS_{60}$ composite under different processing temperatures at stresses ranging from 0 to 150 kPa in successive stretching. The stretching and releasing rates were 0.05 and 1 N·s$^{-1}$, respectively.

**Supplementary Note 12:** To gain an in-depth understanding of the effect of processing temperature on processing performance, we used temperature-dependent rheology techniques to investigate the mechanical behavior of the composites. The elastic modulus of the composite underwent a significant decrease from 88 to 76 and 69 kPa, corresponding to a reduction in viscosity from $1.5\times10^8$ to $1.3\times10^8$ and $1.2\times10^8$ Pa·s with a slight increase in temperature from 30 to 45 and 60 °C (Supplementary Fig. 23a, b). The significant decrease in modulus and viscosities could be attributed to the increased temperature weakening the hydrogen bonding interactions between PS-COOH and PEI[14]. This result was also supported by the stress–strain curves, i.e., the tensile strength decreased to one-third of its original value as the processing temperature increased from 30 to 60 °C (Supplementary Fig. 23c). Furthermore, under the description of creep tests, we found that the strains of the $PEI_{60k}$-$PS_{60}$ composite increased by 3.9%, 8.4%, and 10.7% at processing temperatures of 30, 45, and 60 °C

(Supplementary Fig. 23d), respectively, indicating that the processing temperature significantly increased the chain slippage of the polymer[15]. Correspondingly, the increase in strain rate of the composite with the increase of processing temperature in the tensile cycles of loading and releasing stresses also confirmed these results (Supplementary Fig. 23e). Similar to the mechanism of water-assisted processing of supramolecular composites, the increase in temperature can reduce the mechanical strength and viscosity of the composite, increase the slippage of polymer chains, and promote the migration of colloids under shearing, thereby effectively improving the processability of the composite.

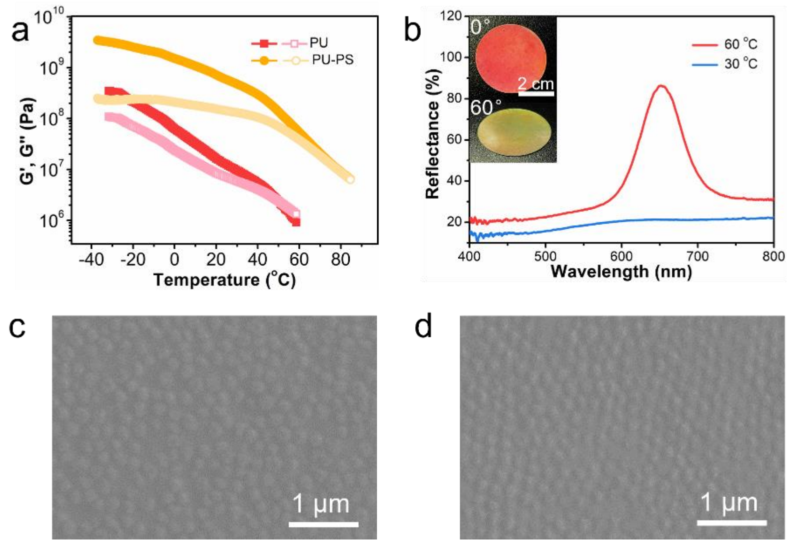


**Supplementary Fig. 24. a** Temperature dependence of the storage modulus G' and loss modulus G'' of the PU ($M_w$: 10 kDa) and PU-PS composite constructed from PU and PS-COOH ($\varphi_{PS}$: 50%). The dynamic moduli were determined by dynamic mechanical analyses (DMA). DMA test was performed on a Diamond Dynamic Mechanical Analyzer (PerkinElmer Instruments). The test was conducted under the tension mode with 1 Hz frequency in the temperature range from -20 to 100 °C at a heating rate of 3 °C·min$^{-1}$. **b** Reflection spectra of PU-PS films at processing temperatures of 30 °C and 60 °C. Insets: the corresponding photographs at a processing temperature of 60 °C with viewing angles of 0° and 60° (sample diameter: 4.0 cm). **c**, **d** SEM images of the PU-PS film at processing temperatures of 30 °C and 60 °C.

**Supplementary Note 13:** To validate the generality of the effect of processing temperature on the processing performance of supramolecular composite, we selected a synthetic linear polyurethane (PU) and PS-COOH to construct a PU-PS

supramolecular composite, in which the urethane groups and carboxylic acid could form hydrogen bonding. The PU was synthesized according to a literature method[16]. The DMA results showed that the introduction of PS-COOH increased the modulus of the polymer by an order of magnitude, which significantly increased the processing difficulty of the composite film (Supplementary Fig. 24a). Therefore, the processed PU-PS films at room temperature cannot generate visible structural color (Supplementary Fig. 24b). In contrast, when the processing temperature was raised to 60 °C, the composite exhibited a transition from the elastic behavior to the viscous state at an oscillation frequency of 1 Hz[15]. The corresponding processed film showed a bright red color with strong angle-dependence (Supplementary Fig. 24b). In addition, by observing SEM images of the colloidal structure of PU-PS films sheared at 30 °C and 60 °C (Supplementary Fig. 24c, d), we found that the increase in processing temperature effectively improved the effect of shear-induced ordering in supramolecular composites.

### 2.12 The versatility of shearing supramolecular composite

**Supplementary Note 14:** To validate the versatility of shearing supramolecular composite, a series of commercially available and synthesized polymers with a wide range of $T_g$ values were selected. Polyvinyl pyrrolidone (PVP, $M_w$ = 40 kDa) and polyacrylic acid (PAA, $M_w$ = 100 kDa) solution were purchased from Sigma Aldrich. Poly(diallyldimethylammonium chloride) solution (PDDA, 20 wt.%, $M_w$ = 400-500 kDa) was purchased from Beijing Innochem Corporation Ltd. The polyborosiloxane (PBSi) was synthesized according to a literature method[17]. The glass transition temperature ($T_g$) of these polymers was measured by differential scanning calorimeter (DSC) (Supplementary Fig. 25).

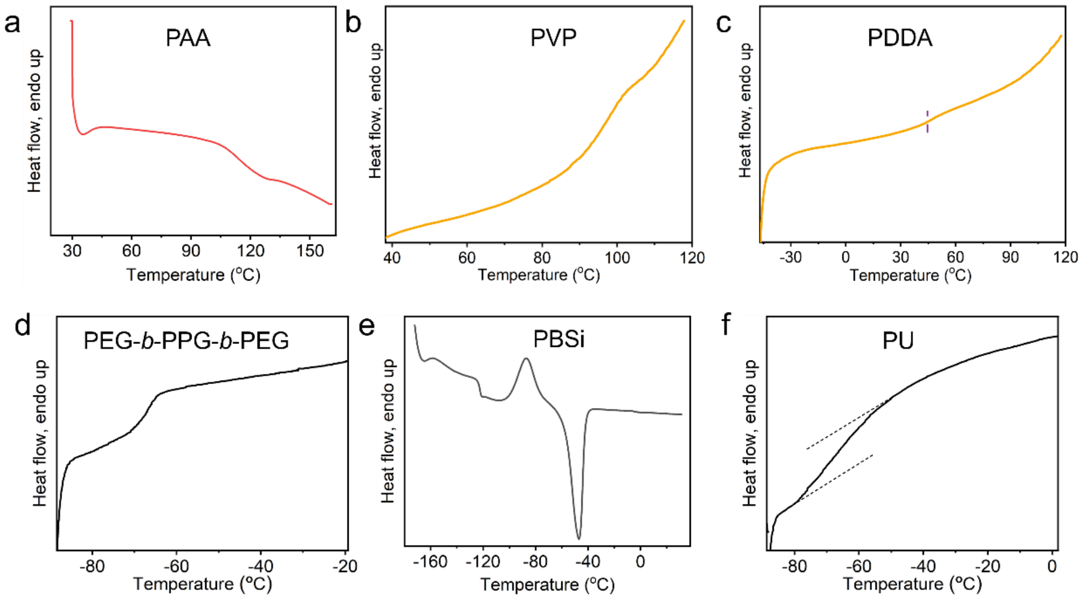


**Supplementary Fig. 25. a-f** DSC curves of PAA ($M_w$: 100 kDa), PVP ($M_w$: 40 kDa), PDDA ($M_w$: ~ 400-500 kDa), PEG-*b*-PPG-*b*-PEG ($M_n$: 14.6 kDa), PU ($M_w$: ~ 10 kDa), and PBSi. DSC measurements were carried out in an $N_2$ atmosphere using a STA449F3 thermal analyzer (Germany) with heating and cooling ramps at 10 °C·min$^{-1}$. The glass transition temperature ($T_g$) of the sample was measured by the inflection point of the second heat in the DSC curves.

These polymers can form supramolecular composites with typical colloids such as PS-COOH or silica ($SiO_2$) colloids. Taking the composite film constructed from PAA ($M_w$: 100 kDa) and PS-COOH, for example, a series of results show that the composite film can be processed into a film with finely colloidal arrangements and outstanding color at room temperature (Supplementary Fig. 26). In addition, significant peaks were observed in the reflection spectra of composite films constructed from other commonly-used polymers and typical PS-COOH or $SiO_2$ colloids, indicating the broad applicability and universality of the shearing supramolecular composite method (Supplementary Figs. 27, 28).

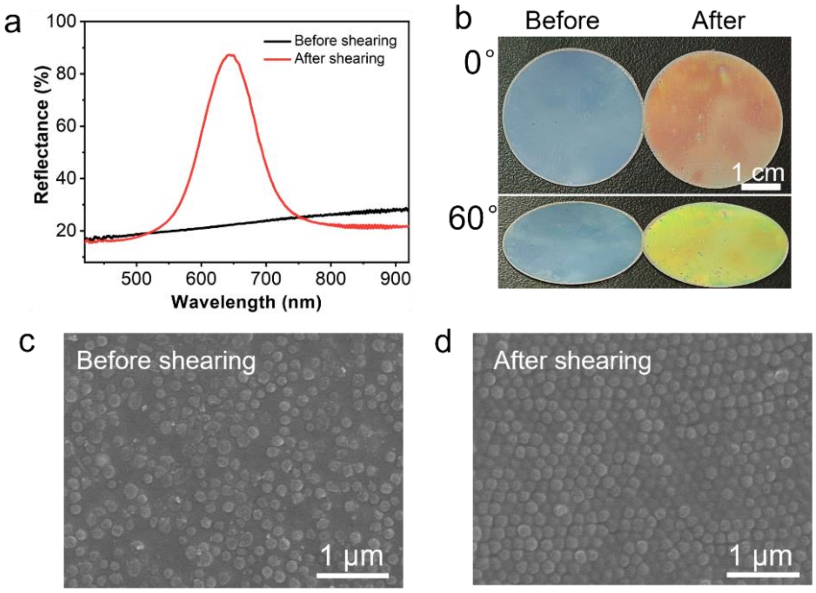


**Supplementary Fig. 26. a** Reflection spectra of the composite films constructed from PAA ($M_w$: 100 kDa) and PS-COOH ($\varphi_{PS}$: 50%) before and after shearing treatments. **b** Photographs of the composite films constructed from PAA ($M_w$: 100 kDa) and PS-COOH ($\varphi_{PS}$: 50%) before and after shearing treatments at viewing angles of 0°and 60° (sample diameter: 3.4 cm). **c, d** SEM images of the composite film constructed from PAA and PS-COOH ($\varphi_{PS}$: 50%) before and after shearing treatments.

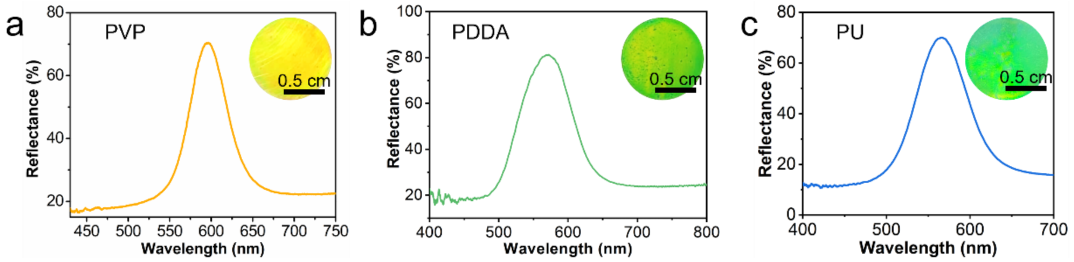


**Supplementary Fig. 27. a-c** Reflection spectra of the composite films constructed from PVP ($M_w$: 40 kDa), PDDA ($M_w$: ~ 400-500 kDa), and PU ($M_w$: 10 kDa) with PS-COOH ($\varphi_{PS}$: 50%) after shearing treatment. Insets in (**a-c**): the corresponding photographs at viewing angle of 0°(sample diameter from left to right: 1.5, 2.0 and 2.0 cm.)

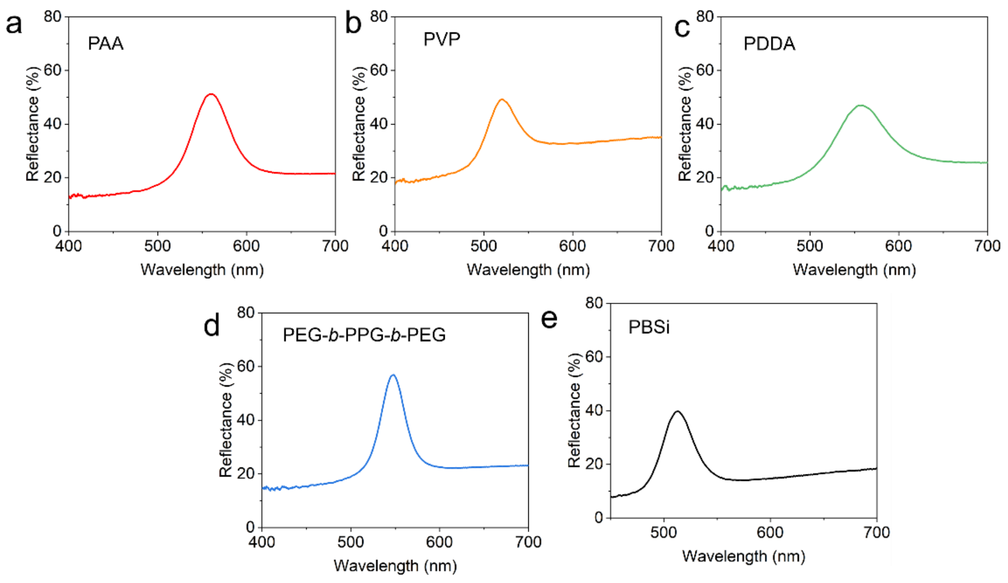


**Supplementary Fig. 28. a-e** Reflection spectra of the composite films constructed from PAA ($M_w$: 100 kDa), PVP ($M_w$: 40 kDa), PDDA ($M_w$: ~ 400-500 kDa), PEG-*b*-PPG-*b*-PEG ($M_w$: 14.6 kDa), and PBSi with $SiO_2$ ($\varphi_{SiO2}$: 50%) after shearing treatment.

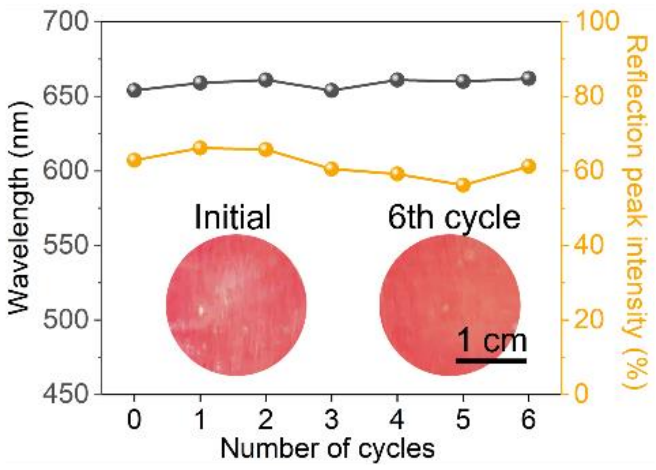


**Supplementary Fig. 29.** Reflection wavelength and peak intensity of SCCFs after sufficient shearing treatments. Insets: corresponding photographs of the initial and cyclic 6th SCCFs.

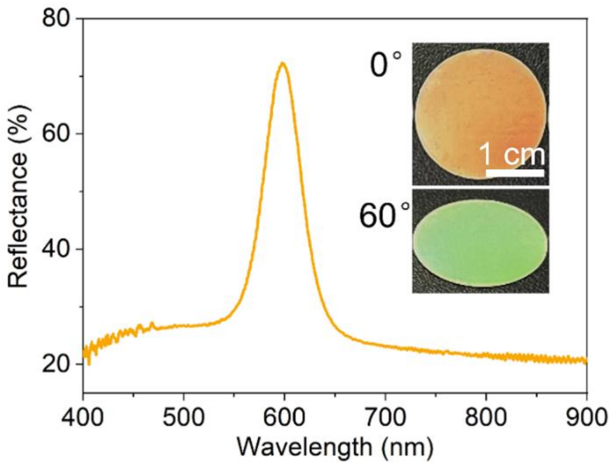


**Supplementary Fig. 30.** Reflection spectrum and photographs (insets) of SCCF after 3 months of storage at room temperature at viewing angles of 0° and 60° (sample diameter: 2.2 cm).

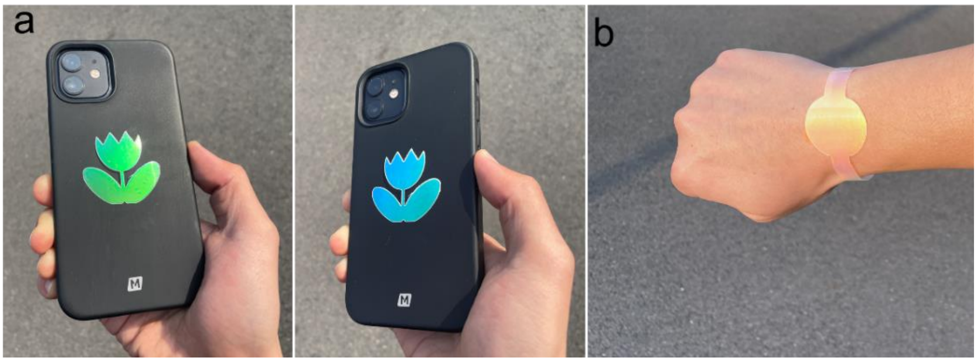


**Supplementary Fig. 31. a, b** Applications of flexible SCCF as decoration of phone cases and stylish wristbands. Experiments were repeated three times independently with similar results. Informed consent has been provided.

## 3. Supplementary Tables

**Supplementary Table 1.** Refractive indices of the matrix polymer and colloid of SCCFs

| Reference | Refractive index of colloid | Refractive index of matrix polymer | Refractive index contrast |
|---|---|---|---|
| This work | 1.590 | 1.529 | 0.061 |
| Ref.19 | 1.450 | ~1.48 | ~0.03 |
| Ref.28 | 1.910 | 1.460 | 0.450 |
| Ref.30 | 1.450 | 1.502 | 0.052 |
| Ref.33 | 1.590 | 1.455 | 0.135 |
| Ref.36 | 2.420 | 1.460 | 0.960 |
| Ref.37 | 1.590 | 1.479 | 0.111 |
| Ref.38 | 1.590 | 1.436 | 0.154 |
| Ref.39 | 1.590 | 1.436 | 0.154 |
| Ref.43 | 1.590 | 1.479 | 0.111 |

**Supplementary Table 2**. Comparative analysis of strategic advantages

| Strategies / Comparative basis | Shearing force (supramolecular composites) | Shearing force (colloids with core-interlayer-shell structures) | Capillary force-induced colloidal assembly | Solvation force-induced colloidal assembly | Magnetic force-induced colloidal arrangement |
|---|---|---|---|---|---|
| **Materials** | Commercial polymers and commonly-used colloids | Colloids with core-interlayer-shell structures obtained by multi-step polymerization | Commonly-used colloids and commercial polymers or functionalized monomers | Commonly-used colloids and functionalized monomers | Magnetic colloids, e.g., $Fe_3O_4$-based colloids and functionalized monomers |
| **Production efficiency** | Seconds to minutes | Seconds to minutes | Hours to days | Hours | Seconds to minutes |
| **Processing conditions** | Room temperature processing | Generally above room temperature processing | Room temperature processing and sometimes UV curing | Additional UV curing | UV and magnetic fields |
| **Preparation steps** | Compositing colloid with polymer and shearing | Multi-step covalent grafting of polymers to colloids and shearing | Co-assembly of colloids with polymers or self-assembly of colloids and introduction of polymers | Co-assembly of colloids with polymer monomers, infiltration into the gap between two glass slides, and UV curing of monomers | Compositing colloid with polymer monomers, magnetic field-induced colloid assembly, and UV curing of monomers |
| **Production area** | Square meter | Square meter | Square meter | Square centimeter | Square centimeter |
| **Supplementary References** | This work | [5], [18-21] | [2], [3], [22] | [23-25] | [26] |

## 4. Supplementary References


1. Tan, H. et al. Metallosupramolecular photonic elastomers with self-healing capability and angle-independent color. *Adv. Mater.* **31**, e1805496 (2019).
2. Peng, X. et al. Ultrafast self-gelling powder mediates robust wet adhesion to promote healing of gastrointestinal perforations. *Sci. Adv.* **7**, eabe8739 (2021).
3. Li, M. et al. Structure-tunable construction of colloidal photonic composites via kinetically controlled supramolecular crosslinking. *Macromolecules* **55**, 8345-8354 (2022).
4. Chen, Y. et al. Tailoring the alignment of string-like nanoparticle assemblies in a functionalized polymer matrix via steady shear. *RSC Adv.* **7**, 8898-8907 (2017).
5. Zhao, Q. et al. Large-scale ordering of nanoparticles using viscoelastic shear processing. *Nat. Commun.* **7**, 11661 (2016).
6. Finlayson, C.E. et al. 3D bulk ordering in macroscopic solid opaline films by edge-induced rotational shearing. *Adv Mater* **23**, 1540-4 (2011).
7. Finlayson, C. E. et al. 3D bulk ordering in macroscopic solid opaline films by edge-induced rotational shearing. *Adv. Mater.* **23**, 1540-1544 (2011).
8. Snoswell, D. R., Finlayson, C. E., Zhao, Q. & Baumberg, J. J. Real-time measurements of crystallization processes in viscoelastic polymeric photonic crystals. *Phys. Rev. E* **92**, 052315 (2015).
9. Xie, F. et al. Carboxyl-terminated polybutadiene–poly(styrene-co-4-vinylpyridine) supramolecular thermoplastic elastomers and their shape memory behavior. *Macromolecules* **49**, 7322-7330 (2016).
10. Peng, Q. et al. Adhesive coacervates driven by hydrogen-bonding interaction. *Small* **16**, e2004132 (2021).
11. Smith, B. A., Mumby, S. J., Samulski, E. T. & Yu, L. P. Concentration dependence of the diffusion of poly(propylene oxide) in the melt. *Macromolecules* **19**, 470-472 (1986).
12. Li, B., You, W., Peng, L., Huang, X. & Yu, W. Revealing the shear effect on the interfacial layer in polymer nanocomposites through nanofiber reorientation. *Macromolecules* **56**, 3050-3063 (2023).
13. Gong, K., Hou, L. & Wu, P. Hydrogen-bonding affords sustainable plastics with ultrahigh robustness and water-assisted arbitrarily shape engineering. *Adv.*

*Mater.* **34**, 2201065 (2022).

14. Chen, S. et al. Hydrogen-bonded supramolecular polymer adhesives: straightforward synthesis and strong substrate interaction. *Angew. Chem. Int. Ed.* **61**, e202203876 (2022).
15. Li, X. et al. Thermoreversible supramolecular networks from poly(trimethylene carbonate) synthesized by condensation with triuret and tetrauret. *Macromolecules* **52**, 6585-6599 (2019).
16. Li, M. et al. Fluorescent metallosupramolecular elastomers for fast and ultrasensitive humidity sensing. *ACS Appl. Mater. Interfaces* **12**, 39665-39673 (2020).
17. Wu, T. & Chen, B. Synthesis of multiwalled carbon nanotube-reinforced polyborosiloxane nanocomposites with mechanically adaptive and self-Healing capabilities for flexible conductors. *ACS Appl. Mater. Interfaces* **8**, 24071-24078 (2016).
18. Li, H., Wu, P., Zhao, G., Guo, J. & Wang, C. Fabrication of industrial-level polymer photonic crystal films at ambient temperature based on uniform core/shell colloidal particles. *J. Colloid Interface Sci.* **584**, 145-153 (2021).
19. Li, H. et al. Polychrome photonic crystal stickers with thermochromic switchable colors for anti-counterfeiting and information encryption. *Chem. Eng. J.* **426**, 130683 (2021).
20. Huang, H. et al. Gecko-inspired smart photonic crystal films with versatile color and brightness variation for smart windows. *Chemical Engineering Journal* **429**(2022).
21. Huang, H. et al. Butterfly-inspired tri-state photonic crystal composite film for multilevel information encryption and anti-counterfeiting. *Adv. Mater*. **35**, 2211117 (2023).
22. He, Y., Liu, L., Fu, Q. & Ge, J. Precise assembly of highly crystalline colloidal photonic crystals inside the polyester yarns: a spray coating synthesis for breathable and durable fabrics with saturated structural colors. *Adv. Funct. Mater.* **32**, 2200330 (2022).
23. Wu, Y., Wang, Y., Zhang, S. & Wu, S. Artificial chameleon skin with super-sensitive thermal and mechanochromic response. *ACS Nano* **15**, 15720-15729 (2021).
24. Lee, G. H. et al. Chameleon-inspired mechanochromic photonic films

composed of non-close-packed colloidal arrays. *ACS Nano* **11**, 11350-11357 (2017).

25. Yang, Q. et al. Large-scale production of high-quality elastic structural color films based on hydrogen bond and colloidal charge co-driven silica microsphere self-assembly. *Chem. Eng. J.* **455**, 140591 (2023).

26. Xie, Y. et al. Bistable and reconfigurable photonic crystals-electroactive shape memory polymer nanocomposite for ink-free rewritable paper. *Adv. Funct. Mater.* **28**, 1802430 (2018).
